# Supplementary material for: Analysis of morphological, morphokinetics, cell-free DNA, microRNAs parameters to predict aneuploidy status of embryos
Source: PLoS One. 2026 Apr 2;21(4):e0344795. doi: 10.1371/journal.pone.0344795 (PMC13046167; doi:10.1371/journal.pone.0344795)
Supplement: Attachment — Submitted filename: Rebuttal Letter (1).docx [file pone.0344795.s002.docx]

30th December 2025

Dear Reviewer/Editorial Team,

Thank you for the opportunity to revise our manuscript and for the constructive feedback from the reviewers. We greatly appreciate their recognition of the innovative nature of our non-invasive approach for embryo assessment. Their detailed comments have been invaluable in improving the clarity and reproducibility of our work.

We have carefully addressed each point raised by the editor and reviewers. All revisions are clearly tracked in the uploaded “Revised Manuscript with Track Changes” file, and a clean version is also provided for your convenience. We have ensured full compliance with PLOS ONE formatting, enhanced methodological transparency, and improved statistical and interpretive accuracy.

Below is a point-by-point summary of our responses and revisions.

**Response to Editor Requirements**

**PLOS ONE Style Requirements, Data Availability & Sharing, Supporting Information Captions, Identifying Data**

**Response:**

Thank you for the kind input, our manuscript has been reformatted using official PLOS ONE templates. All file names have been standardized per journal guidelines. All identifiable patient data (names, specific dates, IDs) have been removed from the Supporting Information files. Due to ethical restrictions under our institutional review board approval (KET-1274/UN2.F1/ETIK/PPM.00.02/2021), which protects the privacy of IVF patients, data cannot be made publicly available prior to acceptance. We have updated the Data Availability statement accordingly and confirm that de-identified datasets will be deposited in Figshare upon acceptance. Complete captions have been added at the end of the manuscript. All in-text citations have been updated accordingly. All columns containing personal or identifying information have been deleted. No decimal ages, specific exam dates, or contact details remain. The files are fully compliant with PLOS ONE anonymization standards.

**Reviewer Citation Suggestions**

**Response:**

We have reviewed all suggested references and incorporated relevant recent literature into the manuscript.

1. Is the manuscript technically sound, and do the data support the conclusions?

The manuscript must describe a technically sound piece of scientific research with data that supports the conclusions. Experiments must have been conducted rigorously, with appropriate controls, replication, and sample sizes. The conclusions must be drawn appropriately based on the data presented.

Reviewer #1: Yes

Reviewer #2: Partly

**Response:**

We thank Reviewer 1 and 2 for acknowledging our work.

2. Has the statistical analysis been performed appropriately and rigorously?

Reviewer #1: Yes

Reviewer #2: Yes

**Response:**

We thank Reviewer 1 and 2 for acknowledging our work.

3. Have the authors made all data underlying the findings in their manuscript fully available?

The PLOS Data policy requires authors to make all data underlying the findings described in their manuscript fully available without restriction, with rare exception (please refer to the Data Availability Statement in the manuscript PDF file). The data should be provided as part of the manuscript or its supporting information, or deposited to a public repository. For example, in addition to summary statistics, the data points behind means, medians and variance measures should be available. If there are restrictions on publicly sharing data—e.g. participant privacy or use of data from a third party—those must be specified.

Reviewer #1: Yes

Reviewer #2: No

**Response:**

We have made adjustments regarding our data availability. Data will be available after manuscript acceptance. Data will be available in the public repository with links provided in our manuscript.

4. Is the manuscript presented in an intelligible fashion and written in standard English?

PLOS ONE does not copyedit accepted manuscripts, so the language in submitted articles must be clear, correct, and unambiguous. Any typographical or grammatical errors should be corrected at revision, so please note any specific errors here.

Reviewer #1: No

Reviewer #2: No

**Response:**

Thank you for the kind feedback, we have revised the entire manuscript, particularly the discussion, has been professionally edited to improve grammar, sentence flow, and readability while reducing wordiness.

5. Review Comments to the Author

Please use the space provided to explain your answers to the questions above. You may also include additional comments for the author, including concerns about dual publication, research ethics, or publication ethics. (Please upload your review as an attachment if it exceeds 20,000 characters)

Reviewer #1: The manuscript addresses the “Analysis of Morphological, Morphokinetics, Cell Free DNAs, microRNAs Parameters to Predict Aneuploidy Status of Embryos”.

The research conducted is solid and contributes to your area of interest. However, I advise a review of the English, especially in the Discussion.

**Response:**

Thank you for the kind feedback, we have revised the entire manuscript, particularly the discussion, has been professionally edited to improve grammar, sentence flow, and readability while reducing wordiness.

**Reviewer 1:**

The conclusion does not exactly reflect the statistical results presented, or the presentation of the statistical data is somewhat confusing, with too much data. I suggest one or more graphs showing the main statistical data found, such as the ROC curve and AUC.

**Response:**

The Conclusion has been rewritten to strictly reflect the logistic regression outcomes. We have simplified tables, added a bridging paragraph explaining the ROC analysis, and included a new ROC figure for clarity.

**Reviewer 1:**

On page 12, it would be better to explain the meaning of OR and CI in (OR 1.48; 95% CI 0.73–3.02, p = 0.18) to make it clearer to the reader. The first time they appear in the text.

**Response:**

On first mention, we now spell out in full: “odds ratio (OR) 1.48, 95% confidence interval (CI) 0.73–3.02, p = 0.18,” with parenthetical explanations added for clarity.

**Reviewer 1:**

Tables 1 and 2 need better formatting.

**Response:**

Tables 1 and 2 have been reformatted for improved readability and alignment with journal standards.

**Reviewer 1:**

In Table 2, all scores are 0 or 1. Why does NiPGT have scores of 0 and 2?

**Response:**

The scoring rationale is now clearly explained in the Methods (Cell-free DNA section): ni-PGT-A = 0 for aneuploid, 2 for euploid (>80% mosaicism threshold via Bluefuse). This weighting reflects its strong predictive power (OR = 286.98) in the multivariate model.

**Reviewer 1:**

It is unclear what the scores in Table 2 were used for.

**Response:**

Table 2 now explicitly presents the clinically applicable scoring system derived from the logistic regression coefficients in Table 1. This system allows embryologists to assign simple point values (0, 1, or 2) based on expansion category, ni-PGT-A result, and miRNA-191 expression, facilitating practical, non-invasive ploidy prediction.

**Reviewer #2:** The manuscript addresses a relevant and contemporary topic, proposing non-invasive parameters for the prediction of embryonic euploidy. However, the current version presents methodological, statistical, and conceptual weaknesses that compromise the scientific robustness of the study.

**Response:**

We are grateful for Reviewer 2’s thorough and insightful comments, which have greatly strengthened the methodological rigor of our manuscript.

**Reviewer 2:**

Main general points requiring revision:

- Revision of scientific language and correction of terminological inaccuracies, such as “cleavage-stage blastocyst,” removing speculative interpretations and redundancies throughout the text, particularly in the introduction and discussion sections.

**Response:**

We have removed inaccurate terms such as “cleavage-stage blastocyst,” eliminated speculative phrasing, and streamlined the Introduction and Discussion to avoid redundancy.

**Reviewer 2:**

- Update of the bibliographic foundation, incorporating more recent studies and including critical comparisons with the literature.

**Response:**

Recent relevant literature has been added, and critical comparisons are now integrated into the Discussion to better contextualize our findings.

**Reviewer 2:**

- Clearly present appropriate control for confounding variables, such as maternal/paternal age of the embryos analyzed and semen quality—factors relevant to expected aneuploidies and potentially compromising causal interpretation.

**Response:**

We have added a dedicated paragraph in the Discussion acknowledging the potential influence of maternal/paternal age and semen quality on aneuploidy risk. While our model focused on embryo-specific markers, we recognize these as important confounders for future validation studies.

**Reviewer 2:**

- Revise the statistical analysis to consider intra-individual dependence for embryos from the same patients (couple), employing appropriate statistical models, and clearly report the number of couples included in the study.

**Response:**

We appreciate the reviewer’s comment. In this study, embryos were analyzed as independent analytical units, as the primary objective was to evaluate embryo-level characteristics (morphology and chromosomal status) rather than patient-level outcomes. Therefore, the analysis was not stratified by individual patient or couple background. This approach is consistent with several previous embryo-based studies in reproductive medicine. Nevertheless, we have clarified the number of couples included in the study in the revised manuscript for transparency.

**Reviewer 2:**

- Revise the statistical presentation by standardizing notation and p-values, replacing “p = 0.000” with corresponding non-zero values.

**Response:**

All p-values are now reported appropriately (“p < 0.001” instead of “p = 0.000”), and notation has been standardized throughout.

**Reviewer 2:**

- Regarding non-invasive genetic analysis:

The cell-free DNA methodology was not sufficiently described to allow critical evaluation or independent replication. The manuscript lacks information about the extraction and quantification techniques for cfDNA, as well as the volume and whether the culture medium used for miRNA analysis was partitioned. Moreover, the discussion does not quantitatively compare the findings with recent literature, merely reiterating previous studies without critically integrating the authors’ own results. It is recommended that the authors provide a detailed description of the cell-free DNA protocol, including analyzed volume, detection method, performing laboratory, classification parameters, and concordance rate with invasive PGT-A, as well as rewrite the discussion to contextualize the observed performance in light of published studies.

**Response:**

We have expanded the Methods section to include detailed cfDNA extraction and quantification protocols, volume of medium used (20 µL), laboratory procedures and classification parameters (Bluefuse 2.0, >80% mosaicism threshold). The Discussion now quantitatively compares our findings with recent literature. miRNA Methodology & Interpretation The Methods now specify miRNA extraction, volume of medium (20 µL), separate from cfDNA analysis, and temporal standardization of collection (Day 5 post-insemination).

**Reviewer 2:**

- Regarding miRNA expression:

The findings related to miRNA-191 and miRNA-372 are potentially interesting; however, the current methodological description is insufficient to assess reproducibility and experimental validity of the presented results. The manuscript fails to report the extraction, quantification, normalization, or quality control methods used for the samples, as well as the culture medium volume and whether it was shared with cfDNA analysis. Furthermore, there is no mention of temporal standardization of collections—a critical factor in gene expression and miRNA studies, since culture time and conditions directly influence genetic material release into the medium.

**Response:**

We appreciate the reviewer’s valuable comments. The manuscript has been revised to clearly describe the sample extraction, quantification, normalization, and quality control procedures. We have also added details regarding the culture medium volume and clarified whether the same medium was used for cfDNA analysis. In addition, temporal standardization of sample collection, including culture duration and conditions, has now been explicitly stated, addressing its importance in gene expression and miRNA analyses.

**Reviewer 2:**

The study also does not correlate miRNA levels with clinical outcomes such as implantation, pregnancy, or live birth rates, although the discussion presents such associations speculatively, unsupported by the generated data. This constitutes extrapolation and reduces the interpretive strength of the results. It is recommended that the authors provide detailed descriptions of miRNA extraction, quantification, and normalization methods, including analyzed volume, specify whether the culture medium was shared with cfDNA analysis, and report collection time standardization. The discussion should also be adjusted to restrict interpretations to effectively tested correlations and remove extrapolations to unassessed clinical outcomes.

**Response:**

The Discussion has been revised to avoid extrapolation to clinical outcomes not assessed in this study (e.g., implantation, live birth). Claims are now strictly limited to correlations supported by our data.

**Reviewer 2:**

- Regarding morphokinetic analysis:

The “expansion grade” parameter was included in the predictive model for euploidy without demonstrating a statistically significant correlation in bivariate analyses or support from explicit kinetic measures, such as blastulation time. Although the literature cites expansion as a potential marker of embryonic competence, in the present study its inclusion appears exploratory and not methodologically justified. It is recommended to reassess the relevance of this variable or explicitly discuss its correlation, possible limitations, or indeed, the absence of a direct correlation.

**Response:**

Expansion grade: We have clarified its inclusion based on logistic regression results (OR = 8.271, p = 0.006) and added a discussion of its biological plausibility and limitations. Morphological parameters (ICM/TE): The Discussion now accurately reflects our findings, no significant association with ploidy was found. We have removed unsupported claims of correlation.

**Reviewer 2:**

- Regarding morphological analysis:

The presented results show no statistically significant association between morphological parameters (ICM, TE, and cleavage stage) and embryonic euploidy status (p > 0.05). Nevertheless, the discussion describes these findings as if there were a positive relationship between embryonic quality and euploidy, which is not supported by the study’s own data. This inconsistency between results and interpretation compromises the scientific coherence of the manuscript.

It is recommended that the authors revise the discussion, removing claims of associations not empirically demonstrated, and reformulate the text to accurately reflect the actual findings. Interpretation should be limited to variables that were truly significant in this study, avoiding generalizations or causal inferences not supported by evidence or derived from unrelated literature.

**Response:**

We revised our discussion as the results of bivariate analysis demonstrated no significant association between ICM morphology (Odd Ratio (OR) 1.48; 95% Confidence Interval (CI) 0.73–3.02, p = 0.18) and TE morphology (OR 1.12; 95% CI 0.54–2.33, p = 0.444) with the chromosomal status of embryos. To prevent any ambiguity, the discussion has been revised to explicitly reflect the non-significant findings, and all statements implying associations not supported by our data have been removed. The current interpretation is now strictly aligned with the statistical results of this study, without extrapolation or causal inference beyond the presented evidence.

**Reviewer 2:**

- Regarding the conclusion:

The study did not employ machine learning techniques, but rather classical logistic regression with ROC analysis. Therefore, the use of the term “machine learning” is conceptually inconsistent and methodologically unsupported. It is recommended that the authors correct this inconsistency by accurately describing the development of an exploratory multivariate regression model, rather than a machine learning algorithm. If the predictive approach is to be maintained, the authors must implement, test, and validate an actual supervised learning model with independent data. Otherwise, the conclusion should be reformulated to reflect the true scope of the study.

**Response:**

We acknowledge the inconsistency and have revised the Conclusion to accurately describe our model as a multivariate logistic regression model, not a machine learning algorithm. All references to machine learning have been removed.

We believe these revisions have substantially strengthened the manuscript in terms of methodological transparency, statistical rigor, and interpretive accuracy. We are grateful for the reviewers’ time and expertise and hope the revised version meets the journal’s standards for publication.

With kind regards,

Dr. Achmad Kemal Harzif, MD, OG (REI)
