# Supplementary material for: Analysis of morphological, morphokinetics, cell-free DNA, microRNAs parameters to predict aneuploidy status of embryos
Source: PLoS One. 2026 Apr 2;21(4):e0344795. doi: 10.1371/journal.pone.0344795 (PMC13046167; doi:10.1371/journal.pone.0344795)
Supplement: Attachment — Submitted filename: Ed new_Reviewer Comments_PlOS One.docx [file pone.0344795.s003.docx]

9 February 2026

Dear Reviewer/Editorial Team,

We sincerely thank you for the thorough evaluation of our manuscript and for the constructive comments that have significantly strengthened the quality and accuracy of our work. We have carefully revised the manuscript in accordance with all suggestions provided.

Here are the following detailed response to each comments of reviewer.

Reviewer #1:

The authors significantly improved the manuscript text and adequately responded to the reviewers' questions and suggestions.
Only one caveat in the text.
In the first line below Table 2, change from "figure below" to "figure 1".

Thank you. We have corrected the reference in the first line below Table 2 from "figure below" to "Figure 1" as highlighted in blue in the manuscript.

Reviewer #2:

The manuscript shows improvement compared with the previous version and partially addresses the reviewers’ requests. However, several important issues remain and must be corrected before further editorial consideration.

- English Language Revision: a further professional revision of scientific English is recommended.
Grammatical errors persist, for example: p.14: “chromosomal abnormalitie”; p.27: “…upregulated miRNA-191 play a role…”.
Terminological inconsistencies are present throughout the manuscript: cell-free DNA vs. cell free DNA; ni-PGT-A vs. niPGT-A.
Problems with reference order and formatting remain: p.26: reference 28 is cited before references 14 and 15; p.27: references 16 and 17 do not follow journal formatting; Reference 29 appears only in the reference list and is not cited in the main text.
The examples above are not exhaustive. A comprehensive linguistic and editorial review of the entire manuscript is required.

We have engaged an English editing service to conduct a thorough linguistic review of the entire manuscript. This addressed all grammatical errors, including the examples noted (p.14: "chromosomal abnormalitie" corrected to "chromosomal abnormalities"; p.27: "upregulated miRNA-191 play a role" revised to "upregulated miRNA-191 plays a role") as highlighted in blue in the revised manuscript. Terminological inconsistencies have been standardized throughout: "cell-free DNA" is now used consistently (hyphenated); "niPGT-A" is uniformly formatted without hyphens or spaces as per journal style.

Reference order and formatting issues have been resolved: On p.26, reference 28 now follows 14 and 15 in citation sequence; on p.27, references 16 and 17 conform to journal guidelines. All references were cross-checked in the revised manuscript.

- Inadequate Statistical and Scientific Terminology: the request to revise scientific language remains outstanding.
Incorrect expressions are still present, including: p.20: “p = 0.000”; p.24: “cleavage-stage blastocysts”

Thank you. The original phrasing "p = 0.000" has been corrected to "p < 0.001" to adhere to standard statistical reporting conventions, which avoid exact p-values of zero due to computational precision limits. In the revised manuscript The term "cleavage-stage blastocysts" has been replaced with "cleavage-stage embryos" throughout, as blastocysts refer specifically to the post-compaction stage (typically day 5–6), whereas cleavage-stage embryos encompass earlier divisions up to the morula.

- Use of the Term “Machine Learning”: replacement of this term remains incomplete.
A reference to “machine learning” is still present on p.28. All occurrences must be removed or technically justified.

Thank you for noting the remaining "machine learning" reference on p. 28. We have removed all instances throughout the manuscript, replacing them with appropriate terms. No occurrences remain.

- Discussion and Use of the Literature: the discussion has been expanded with more recent references, which is appropriate. However, extensive discussion of miRNAs that were not investigated in the present study has been added. These sections should be removed or clearly identified as background information only. The Discussion should be restructured to: avoid extrapolation beyond the presented data, reduce redundancy and maintain exclusive focus on the variables analyzed.

Thank you for acknowledging. We have restructured the Discussion to focus solely on the variables analyzed in this study. Extensive sections on miRNAs not investigated here have been removed, with only essential background retained and clearly labeled as such. Redundancy has been minimized, and all extrapolations beyond our presented data have been eliminated.

-On pag. 21 the term ni-PGT-A should be fully defined at its first appearance (p.21)

Thank you. Noninvasive preimplantation genetic testing for aneuploidies (niPGT-A) will be added in the revised manuscript.
